# Evaluating the Specificity of Cognitive Control Deficits in Schizophrenia Using Antisaccades, Functional Magnetic Resonance Imaging, and Healthy Individuals With Poor Cognitive Control

**DOI:** 10.3389/fpsyt.2018.00107

**Published:** 2018-04-11

**Authors:** Amanda L. Rodrigue, David J. Schaeffer, Jordan E. Pierce, Brett A. Clementz, Jennifer E. McDowell

**Affiliations:** Clinical and Cognitive Neuroscience Laboratory, Department of Psychology, University of Georgia, Athens, GA, United States

**Keywords:** schizophrenia, cognitive control, functional magnetic resonance imaging, antisaccades, specificity

## Abstract

Cognitive control impairments in schizophrenia (SZ) can be evaluated using antisaccade tasks and functional magnetic resonance imaging (fMRI). Studies, however, often compare people with SZ to high performing healthy people, making it unclear if antisaccade-related disruptions are specific to the disease or due to generalized deficits in cognitive control. We included two healthy comparison groups in addition to people with SZ: healthy people with high cognitive control (HCC), who represent a more typical comparison group, and healthy people with low cognitive control (LCC), who perform similarly on antisaccade measures as people with SZ. Using two healthy comparison groups may help determine which antisaccade-related deficits are specific to SZ (distinguish SZ from LCC and HCC groups) and which are due to poor cognitive control (distinguish the LCC and SZ groups from the HCC group). People with SZ and healthy people with HCC or LCC performed an antisaccade task during fMRI acquisition. LCC and SZ groups showed under-activation of saccade circuitry. SZ-specific disruptions were observed in the left superior temporal gyrus and insula during error trials (suppression of activation in the SZ group compared to the LCC and HCC group). Differences related to antisaccade errors may distinguish people with SZ from healthy people with LCC.

## Introduction

Cognitive control involves filtering out distracting information in order to perform goal-directed responses, utilizing aspects of attention, inhibition, and working memory. Cognitive control abilities can be assessed using antisaccade tasks. To begin the task, participants fixate on a central target. When a cue appears in a peripheral location in the horizontal plane, they are instructed to direct their glance to the mirror image location (opposite direction, same distance from center) (1); glances toward the cue are errors and considered failures of cognitive control. Antisaccade tasks measure cognitive control because successful performance relies on the several key operations: attention to a visual cue, inhibition of the pre-potent response to look toward the cue when it appears, and the generation of a voluntary response (the glance in the opposite direction), all while engaging working memory for task rules (2, 3). Results of functional magnetic resonance imaging (fMRI) studies show that antisaccade performance is supported by regions distributed throughout the brain [supplementary and frontal eye fields (FEFs), posterior parietal cortex, and subcortical regions] (4–9), although frontal regions like the dlPFC are particularly important given that lesions result in elevated antisaccade error rates (10–13).

Antisaccade tasks have been used as a research tool to evaluate cognitive control disruptions in schizophrenia (SZ) [see (14–17) for reviews]. People with SZ make more antisaccade errors than healthy comparisons and sometimes exhibit slower reaction times (RT) when they perform a correct antisaccade response (5, 8, 18–23). Neural disruptions underlying poor antisaccade performance in SZ (as measured by fMRI) include under-activation of the FEFs, visual regions, basal ganglia, and most consistently the dlPFC when compared to healthy controls (5, 8, 21, 24–27). Under-activation of other frontal regions also is apparent during antisaccade errors, including the anterior cingulate and insula, indicating that additional deficits in error processing may contribute to poor antisaccade performance (28). More importantly, deviations in both behavior and brain circuitry are seen in unaffected first-degree relatives (8, 20, 29, 30), suggesting that these measures serve as possible endophenotypes for the disorder.

While antisaccade-related disruptions in SZ are well-established and their status as endophenotypes seems promising, a majority of studies compare people with SZ to healthy groups who have intact cognitive control (31–33). Antisaccade-related deficits in SZ, therefore, could be due to differences in cognitive ability rather than specific to the disorder. Evidence that this may be the case comes from studies of healthy people who perform just as poorly as people with SZ on antisaccade tasks (34, 35). Healthy individuals who have poor antisaccade performance exhibit similar underlying brain disruptions as people with SZ, including under-activation of frontal regions during both correct and error antisaccade trials (35). These same healthy individuals, however, also exhibit hyper-activation in visual regions, which is not reported in people with SZ. The lack of inclusion of low performing healthy individuals and people with SZ in the same study design makes direct comparisons between the two groups difficult, leaving the question open as to whether antisaccade-related disruptions are specific to SZ.

This study uses two healthy comparison groups: people with intact cognitive control, who are representative of healthy comparison groups commonly used in the literature [referred to here as the high cognitive control group (HCC)], and people who have low cognitive control [referred to here as the low cognitive control group (LCC)] as a more appropriate comparison sample for the SZ group. By including two healthy comparison groups, we aim to isolate antisaccade disruptions that are specific to SZ (i.e., differentiate the SZ group from the LCC and HCC groups) from those that are due to general deficits in cognitive control and are not associated with the psychiatric diagnosis (i.e., differentiate the LCC and SZ groups from the HCC group). Additionally, we evaluate activation associated with correct and error trials, given that distinctions between groups could arise from separable disruptions related to correct responses, error responses, or both.

We hypothesized that the SZ and LCC groups would show similar deficits in antisaccade performance compared to the HCC group. We also hypothesized that disruptions underlying poor performance would manifest as under-activation of brain circuitry, particularly in frontal regions, in both the LCC and the SZ groups, although hyper-activation in visual regions may be solely present in the LCC group.

## Materials and Methods

### Participants

Sample characteristics are described in Table 1. SZ subjects (*N* = 23) were recruited from the community and outpatient facilities in Athens, GA, USA and Augusta, GA USA. Healthy subjects were recruited from the community in Athens, GA, USA. HCC and LCC healthy comparison groups were drawn from a large initial sample (*N* = 235; mean age = 31 years, SD = 11; 53% female) and defined based on a composite score calculated from averaging the *z*-transformed scores of three complex working memory SPAN tasks: reading span, operation span, and symmetry span. Despite the label of “working memory,” these tasks evaluate an individual’s ability to not only maintain information (like typical tasks of working memory) but also maintain information in the face of distracting and irrelevant stimuli, much like tasks involving cognitive control. Furthermore, SPAN tasks have good test–retest reliability (36) and predict performance on both higher order and lower order cognitive control tasks (34, 37), including antisaccades (3, 34, 38). Using established norms (39), comparison subjects with composite scores in the upper quartile (above 75%) were included in the high cognitive control group (HCC; *n* = 21), whereas comparison subjects with composite scores in the lower quartile (below 25%) were included in the LCC group (LCC; *n* = 27). SZ subjects also completed the SPAN tasks. SPAN scores in the SZ and LCC group were not significantly different [*t*(44) = 0.68, *p* = 0.49]. Groups also did not differ in regards to age [*F*(2,68) = 2.0 *p* = 0.14], sex [χ^2^(2, *N* = 71) = 3.4, *p* = 0.17], or handedness [χ^2^(4, *N* = 71) = 2.8, *p* = 0.17].

**Table 1:** Subject characteristics.

	HCC (*n* = 21)	LCC (*n* = 27)	SZ (*n* = 23)
SPAN composite	0.64 (0.31)	−1.35 (0.88)	−1.55 (1.1)
Age (years)	33.6 (12.6)	38.9 (10.5)	39.9 (10.7)
Gender (male)	14	13	9
Handedness (right, left, ambidextrous)	19, 1, 1	25, 3, 0	19, 2, 2
Psychotropic medication			
Unmedicated			6
Anti-psychotic (typical, atypical, both)	–	–	2, 10, 1
Anti-depressant	–	–	5
Benzodiazepines	–	–	2
Polypharmacy	–	–	8

*Cells show numbers for each item except SPAN Composite and Age, which show mean (SD). SPAN data for four participants (one HCC and three LCC) were lost at a later date due to technical difficulties. Polypharmacy was defined as taking more than one psychotropic medication. One SZ subject was taking lithium in addition to antipsychotics and one SZ subject was taking Adderal. HCC = high cognitive control group; LCC = low cognitive control group; SZ = schizophrenia group*.

Participants were administered the Patient or Non-Patient Edition of the Structured Clinical Interview for DSM-IV TR. Exclusion criteria for all groups included substance abuse within the last month and/or substance dependence within the last 6 months. Additional exclusions for comparison subjects included having past personal history of a psychotic or mood disorder, or a first-degree relative with psychosis. All subjects were free from contraindications for MRI (metal in the body and pregnancy) and reported no history of head trauma. All subjects signed consent forms and were paid for their time. Study procedures were reviewed and approved by the University of Georgia Institutional Review Board.

### Antisaccade Task Design

**Figure 1:**
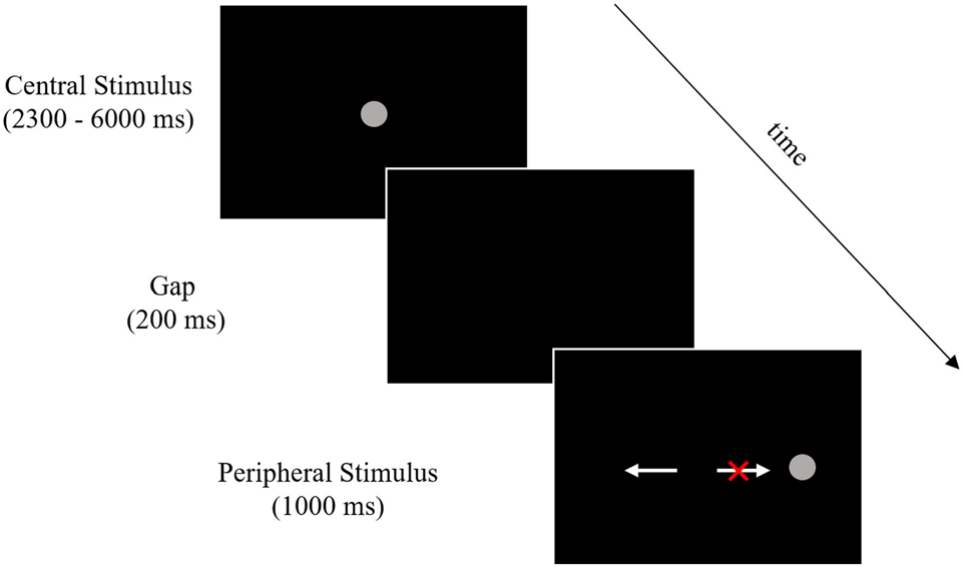
Stimuli and timing. Participants performed 120 trials across two runs of an antisaccade task (60 trials per run). The above image outlines a single trial, which began with a central stimulus. Central stimulus timing was pseudorandomly jittered between 2,300 and 6,000 ms in order to deconvolve the stimulus-related hemodynamic response in the rapid event-related design. A 200-ms gap was then introduced followed by the peripheral stimulus presentation. Participants were given 1,000 ms after the peripheral stimulus onset to execute a glance in the opposite direction. The white arrow pointing to the left indicates a correct response (a glance to the mirror image of the peripheral stimulus) and the white arrow pointing to the right with the red “X” indicates an error response (a glance toward the peripheral stimulus). Arrows and the red X were not present during the task.

Participants performed two runs of antisaccades (60 trials each) in the scanner using an event-related design. Stimuli and task timing are shown in Figure 1. Stimuli were 1.5° gray filled circles presented on a black background. Each trial began with the stimulus located in the center of the screen, followed by its disappearance and reappearance at ±5° or 10° from center in the horizontal plane. Participants were told to look at the stimulus when it was in the center and execute a glance in the opposite direction, same distance from center, when it appeared in one of the four peripheral locations.

### Procedure and Imaging Parameters

MRI data were collected at the Bio Imaging Research Center at the University of Georgia, using a 3T GE Signa MRI (General Electric Medical Systems, Milwaukee, WI, USA) and an eight channel head-coil. Participants were given task instructions before being positioned in the scanner. A high resolution structural scan was conducted to identify the plane of the anterior and posterior commissure (AC–PC) and for later use in preprocessing [T1-weighted 3D FSPGR, repetition time (TR) = 8.1, echo time (TE) = 3.1, flip angle = 20°, field of view (FOV) = 240 mm × 240 mm, matrix size = 256 × 256, 150 axial slices, in-slice resolution = 0.94 mm × 0.94 mm, slice thickness = 1.2 mm]. Following the structural scan, participants completed two functional scans while performing the antisaccade task [T2*-weighted gradient echo EPI sequences, repetition time (TR) = 2,000 ms, TE = 30 ms, flip angle = 90°, FOV = 220 mm × 220 mm, matrix size = 64 × 64, 33 interleaved oblique slices, in-slice resolution = 3.4 mm × 3.4 mm, slice thickness = 4 mm, slice gap = 0 mm, scan time = 5:26, 158 volumes plus 4 initial dummy scans to allow for magnet stabilization]. Participants viewed the stimuli on a screen positioned at their feet (174 cm from the nasion) via a mirror box placed on top of the head coil (16 cm above and in front of the eyes). Stimuli were displayed using Presentation Software (Neurobehavioral Systems, Albany, CA, USA) and eye movements during the scan were recorded using an IView X MRI-LR system with a sampling rate of 60 Hz (SensoMotoric Instruments, Berlin, Germany).

### Analysis

#### Antisaccade Behavior

Eye movement data from the scanner environment were scored using an in-house program generated in MATLAB (The Mathworks Inc., Natick, MA, USA). Antisaccade trials were scored for initial direction (correct or error response) and correct RT (time taken to initiate a correct response from appearance of peripheral cue). Trials with no response, blinks at stimulus onset, anticipatory saccades (faster than 90 ms RT from peripheral stimulus onset or during the gap window), or with insufficient data quality due to loss of pupil tracking were considered unscorable and eliminated from behavioral analysis. Error rate [(number of error trials/total number of scorable trials) × 100] and average correct RT were calculated for each participant. Means were compared with one-way ANOVAs followed by Tukey–Kramer *post hoc* comparisons.

#### Neuroimaging

Imaging analysis was performed with Analysis of Functional NeuroImages (AFNI) (40). Three-dimensional datasets were created from individual DICOM files for each antisaccade run. Preprocessing of functional images included despiking, slice timing correction, registration to a representative volume for movement, alignment of functional data to anatomy, smoothing with a 4-mm full-width at half-maximum Gaussian filter, and scaling each voxel to a mean of 100 as in Camchong et al. (24) and Dyckman et al. (41). One 4D file was created for each subject by concatenating the two preprocessed antisaccade runs. Concatenated time series were analyzed with a generalized least squares time series fit after temporal auto-correlation estimation using the Restricted Maximum Likelihood Model procedure in AFNI [REML; ARMA (1,1)]. The time series fit used a model with separate regressors for correct and error trials that were specific to each subject’s behavioral performance; non-scorable trials were incorporated as a separate regressor to avoid inclusion in the baseline. The model also included regressors for linear, quadratic, and cubic drifts as well motion regressors (3 translational and three rotational) from the alignment step as regressors of no interest.

Group analyses were performed with a whole brain mixed-effects multilevel analysis (MEMA), which accounts for heterogeneity within groups by taking into account the accuracy and precision of estimates from individual parameter estimates. This makes group-level analysis less susceptible to spurious results when the variance in the effect of interest within a group is comparable to the variance across groups or when outliers are present [for details see Chen et al. (42)]. Two MEMA analyses were performed to determine if there were activation differences between each of the three groups: one for correct trials and one for error trials. To avoid false positives, group maps from the MEMA analysis were corrected using a clustering procedure derived from Monte Carlo simulations; the minimum number of voxels that constituted a cluster at a family-wise α = 0.05 was 45.

## Results

### Antisaccade Behavior

**Figure 2:**
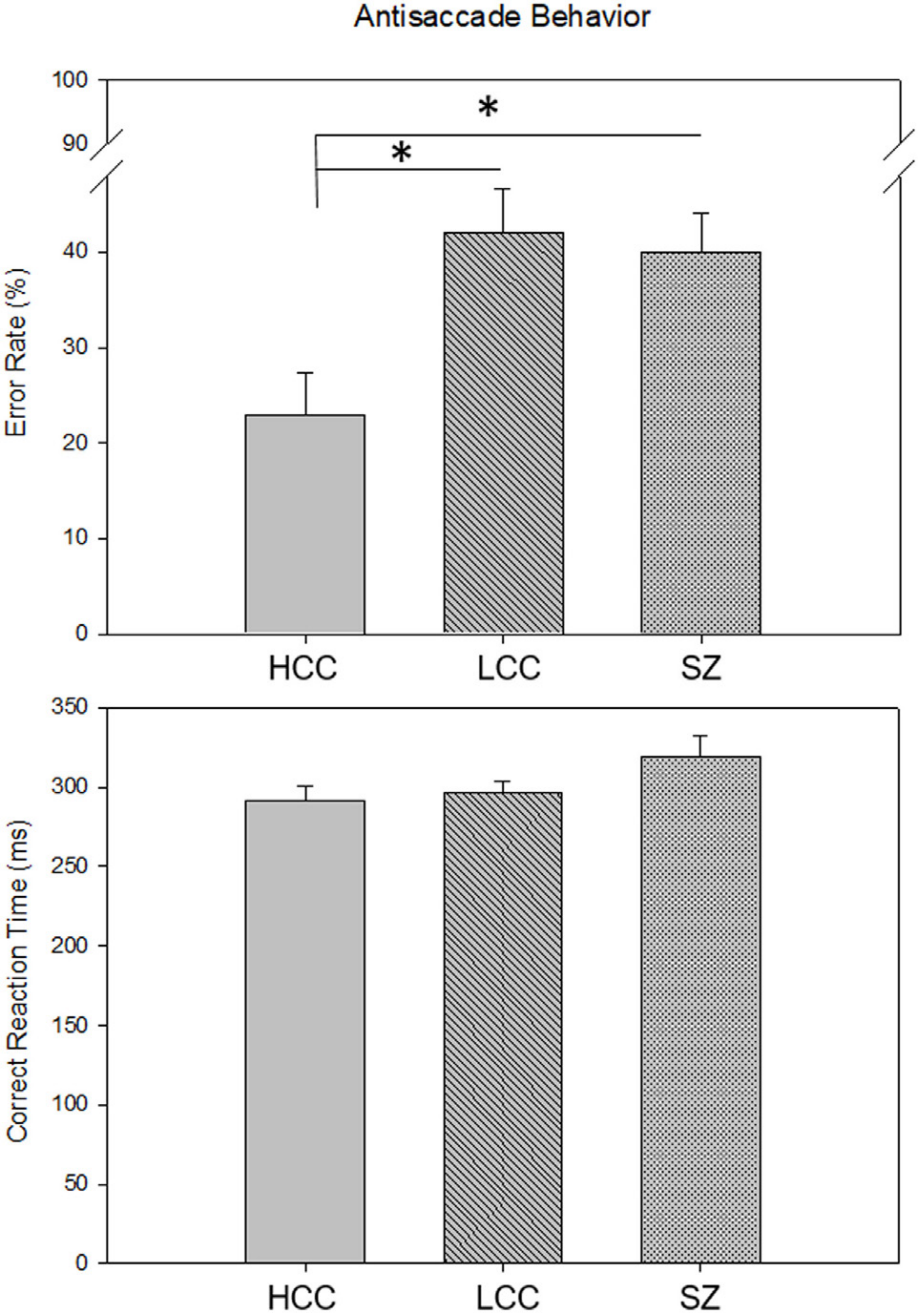
Group Differences in Antisaccade Behavior. Bars show mean (SE) antisaccade error rate (top) and mean (SE) correct reaction time (bottom) for each group. Asterisks indicate significant differences between groups *p* < 0.05 [HCC vs. LCC: *t*(46) = −3.2, *p* = 0.005; HCC vs. SZ: *t*(48) = −2.62, *p* = 0.02]. HCC, high cognitive control group; LCC, low cognitive control group; SZ, schizophrenia group.

Error rates and correct RTs for each group are shown in Figure 2. There were significant differences in error rate among the three groups [*F*(2, 68) = 5.7, *p* = 0.005]. Groups with poor cognitive control (LCC and SZ) had higher error rates than the HCC group (HCC: M = 0.23, SD = 0.19; LCC: M = 0.42, SD = 0.23; SZ: M = 0.39, SD = 0.19). Individuals with SZ were slightly slower at executing correct antisaccade responses than both healthy groups, but this was not statistically significant [*F*(2, 68) = 1.9, *p* = 0.14]. The proportion of scorable trials did not differ across groups (HCC: M = 0.85, SD = 0.13; LCC: M = 0.82, SD = 0.12; SZ: M = 0.85, SD = 0.10; *F*(2,68) = 0.41, *p* = 0.66). The proportion of corrected errors also did not differ across groups indicating that all participants understood the task and performed the task with sufficient motivation [HCC: M = 0.88, SD = 0.12; LCC: M = 0.86, SD = 0.15; SZ: M = 0.85, SD = 0.12; *F*(2,68) = 0.34, *p* = 0.71].

### Neuroimaging

**Figure 3:**
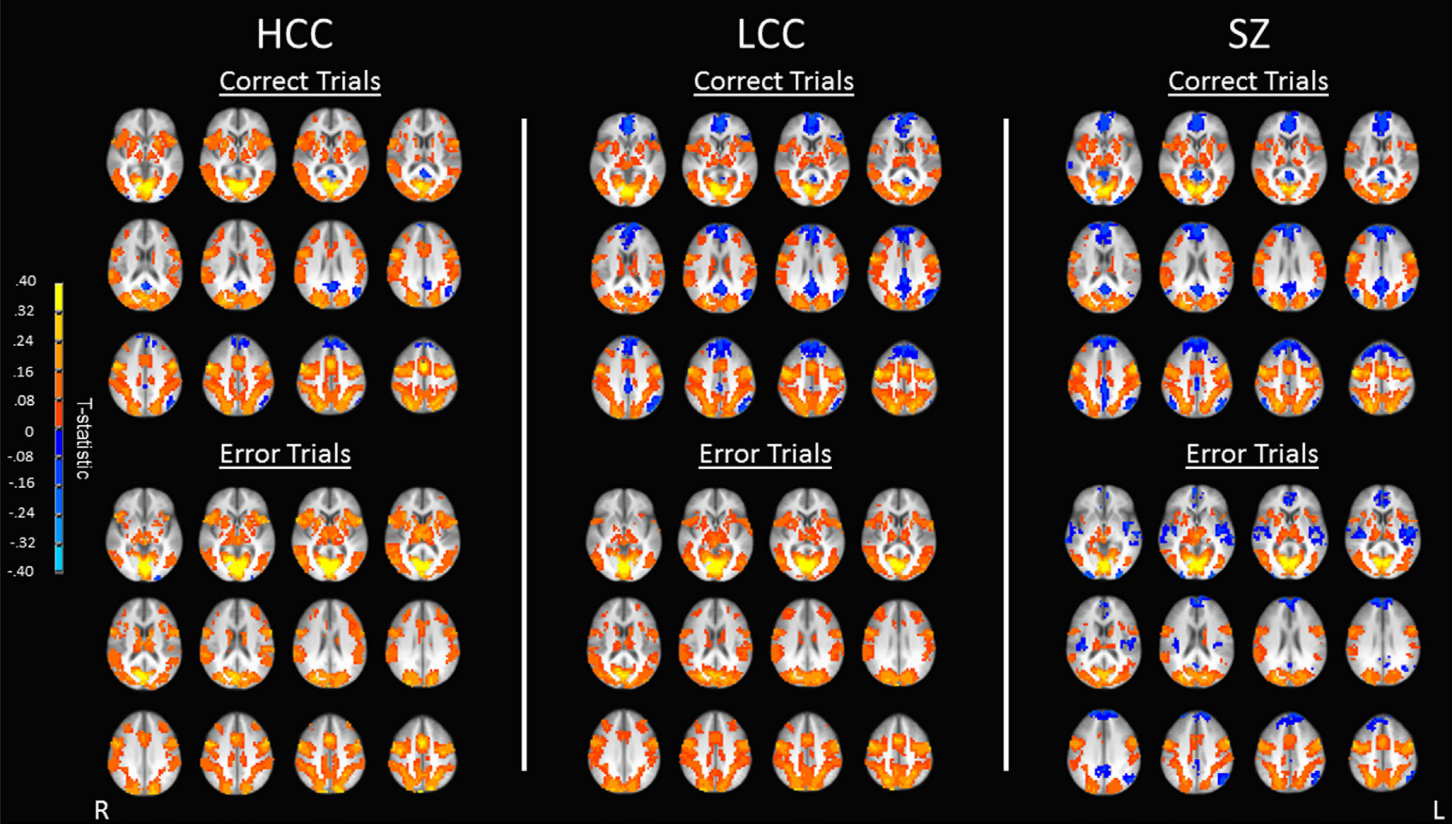
Antisaccade activation for correct and error trials. Maps show activation as measured by BOLD percent signal change for correct trials (top) and error trials (bottom) for each of the three groups (HCC, LCC, SZ). Warm colors indicate positive activation during correct and error trials, cool colors indicate task-induced suppression during correct and error trials. Activations are shown on an anatomical image averaged across all subjects in Talairach space (*z* = 0 to *z* = 44) in radiological orientation (right side shown on the left). HCC, high cognitive control group; LCC, low cognitive control group; SZ, schizophrenia group.

**Figure 4:**
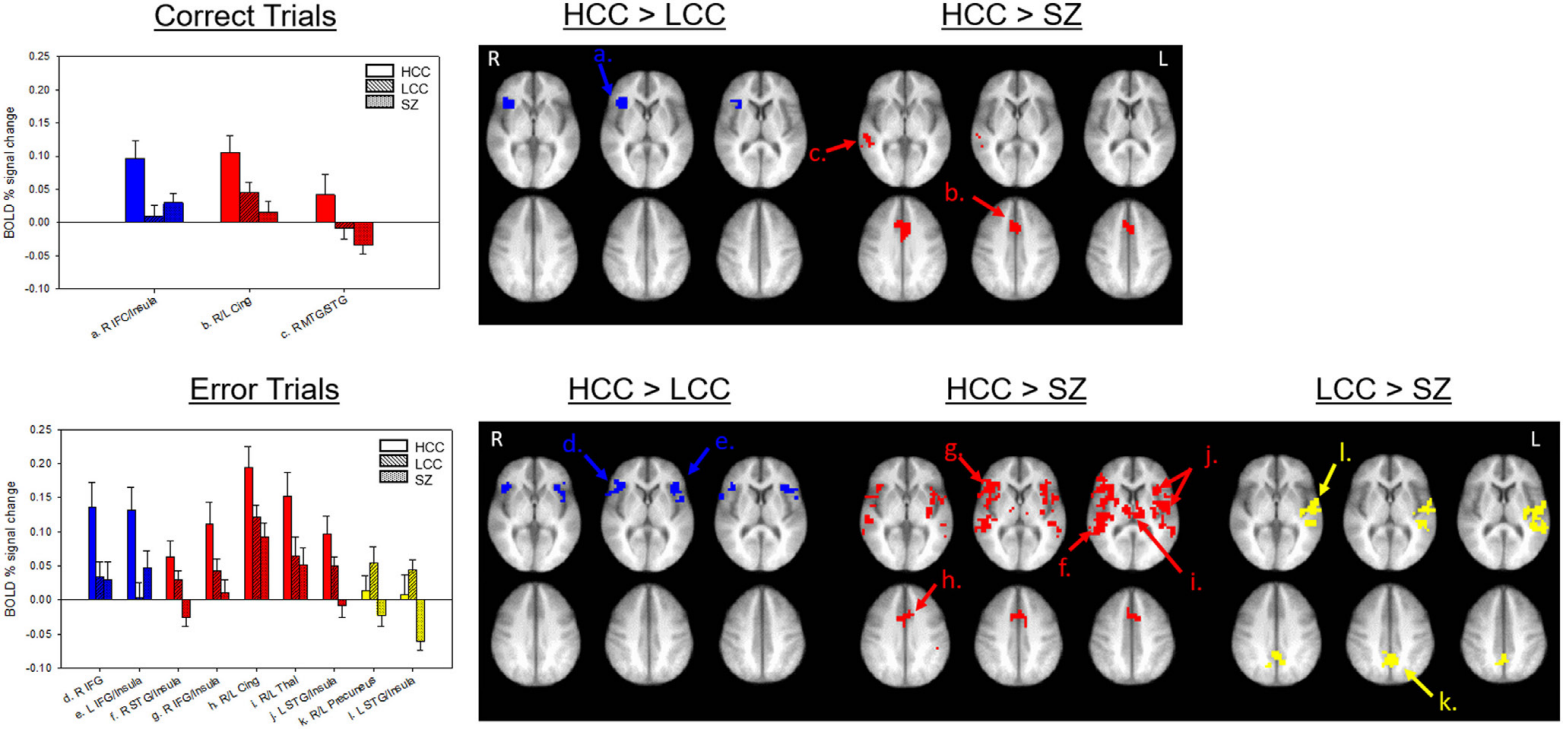
Group-wise differences in antisaccade activation. Top panel shows group-wise differences in antisaccade correct trial activation. Bottom panel shows group-wise differences in antisaccade error trial activation. Bars (SE) show levels of activation for each group in each labeled cluster from Table 2. Clusters are shown on an anatomical image averaged across all subjects in Talairach space (top *z* = 2, 6, 10; bottom *z* = 31, 35, 39) I radiological orientation. Colors indicate in which comparison the cluster was significant. HCC, high cognitive control; LCC, low cognitive control; SZ, schizophrenia; IFG, inferior frontal gyrus; MTG, middle temporal gyrus; STG, superior temporal gyrus.

All groups showed robust BOLD percent signal change in saccade circuitry during correct and error trials (see Figure 3). Groups did not differ in their amount of stimulus-correlated movement in any parameter (see Table S1 in Supplementary Material). BOLD percent signal change during antisaccade correct and error trials was reduced in both the LCC and SZ groups (see Figure 4; Table 2). Both LCC and SZ groups showed significantly lower BOLD percent signal change than the HCC group in inferior frontal gyrus (IFG) and anterior insular regions. In the remaining clusters (for both correct and error trials), the LCC group displayed intermediate levels of BOLD percent signal change between the SZ and HCC group, although there were no significant differences between the HCC and LCC groups in these clusters as determined by the MEMA analysis. The exception was bilateral precuneus and left superior temporal gyrus (STG), where error-related BOLD percent signal change was reduced in the SZ group only. Differences between the SZ and HCC group, however, were only significant for the left STG/insula cluster [*t*(42) = 2.19, *p* = 0.03].

**Table 2:** Mixed-effects multilevel analysis (MEMA) results.

Analysis	Hemi-sphere	Region	*x*	*y*	*z*	Cluster Size (voxels)	Letter in Figure 3
**Correct trials**							
HCC > LCC							
	R	IFG/Insula	35	20	7	45	a
HCC > SZ							
	R/L	Cingulate	0	12	34	88	b
	R	MTG/STG	55	−32	−1	57	c

**Error trials**							
HCC > LCC							
	R	IFG	46	17	9	66	d^1^
	L	IFG/Insula	−44	14	12	82	e^2^
HCC > SZ							
	R	STG/Insula	48	−31	9	149	f
	R	IFG/Insula	42	16	9	140	g^1^
	R/L	Cingulate	0	9	38	74	h
	R/L	Thalamus	−2	−13	14	47	i
	L	STG/Insula	−43	−13	12	221	j^2,3^
LCC > SZ							
	R/L	Precuneus	1	51	35	52	k
	L	STG/Insula	−48	−22	11	176	l^3^

*Significant clusters from the mixed effects multilevel analysis for correct and error antisaccade trials. x, y, z coordinates are in Talairach space and indicate center of mass for each cluster. Some clusters extended beyond a single region, primarily between the insula and temporal regions. Superscript numbers indicate clusters that spatially overlapped with each other among comparisons. HCC, high cognitive control group; LCC, low control group; SZ, schizophrenia group; IFG, inferior frontal gyrus; MTG, middle temporal gyrus; STG, superior temporal gyrus*.

## Discussion

People with SZ exhibit cognitive control deficits, as evidenced by poor antisaccade performance and deviations in circuitry activation as measured by fMRI. To investigate the specificity of these patterns to disease vs. those associated with overall effects of poor cognitive control, we used two healthy comparison groups (HCC and LCC). Results were mostly supportive of a general cognitive control impairment, with many antisaccade deviations shared between the LCC and SZ groups. Both groups had high antisaccade error rates and under-activated key regions of antisaccade circuitry. This under-activation was mostly consistent with previous reports (8, 24, 35, 43), although we did not see hyper-activation of visual regions in the current LCC group (35).

For correct and error trials, under-activations in the LCC and SZ groups were predominant in the IFG, insula, cingulate cortex, and temporal cortex. Overlap of under-activated regions between correct and error trials is expected given that fronto-insular and cingulate cortices comprise a distributed salience network (44) that is activated during multiple stages of cognitive processing: cue presentation (related to initial processing of behaviorally salient cues), task performance (facilitation of attention and task set maintenance), and error-related processing (45). Fronto-insular and cingulate regions also are recruited across tasks requiring responses that involve more than one competing response set (46, 47), like antisaccades, and provide vital information to dorsal attention systems via inputs to the dorsal lateral prefrontal cortex (DLPFC) (45, 48).

Nodes within this salience network are involved in a number of cognitive operations relevant to antisaccade performance. The IFG, particularly in right hemisphere, is involved in inhibitory control as well as action updating and selection when non-dominant responses are required (49–51). Inhibition is important for successful antisaccade performance, as the competing and more dominant response of looking toward the peripheral cue must be suppressed before a “non-dominant” response is generated in the opposite direction. Additionally, the right IFG and insula are part of the ventral attention network, which allows for orientation to salient and behaviorally relevant stimuli as well as volitional redirection of attention (52, 53). Fronto-insular activation is particularly strong for unexpected cues that require reorientation of attention (48). Attentional processes are central to antisaccade performance given the spatial uncertainty of the peripheral cue, the initial requirement to covertly attend to it, followed by switching attention to the opposing location. Under-activation of fronto-insular regions during correct responses may indicate deficits in both inhibition and attentional allocation that may make people with poor cognitive control (LCC and SZ groups) susceptible to higher antisaccade error rates regardless of psychiatric diagnosis.

The salience network, composed of these fronto-insular regions and cingulate cortex, also shows significant activation during error commission and is essential for successful error processing. Network nodes reach peak activation early, before other control regions like the DLPFC (7, 45), indicating that they may feed forward information to top-down portions of saccade circuitry to adapt future behavior. Ham and colleagues (54) suggest a hierarchy of regions that communicate information immediately after an error has occurred, starting with the right insula. The insula then serves as an output hub to the cingulate, which provides error-related signals to dlPFC, resulting in post-error adjustments in behavior (54). Blunted error responses, in the form of fronto-insular and cingulate under-activation, are common in people with SZ and contribute to poor behavioral performance (28, 55–57), although the LCC group showed similar patterns of under-activation in our sample. Both LCC and SZ groups also corrected errors to the same degree as the HCC group, suggesting that groups with poor cognitive control did not have problems detecting errors, but instead, may have had problems using immediate feedback from error commission to establish and implement appropriate task sets, making errors more common. This interpretation seems likely given involvement of fronto-insular and cingulate regions in task set implementation (45).

Although the LCC and SZ groups shared a majority of antisaccade-related deviations, one aspect that distinguished the SZ group from the LCC group was suppression of the left STG and more posterior parts of the insula during antisaccade errors. A similar pattern was observed in the same regions in the right hemisphere, but did not significantly differ from the LCC group. The STG co-activates with, and structurally connects to fronto-insular regions (58–61), making it an important contributor to the functions of the aforementioned brain networks. Additionally, the STG plays a role in maintaining information related to prior outcomes over time in order to assess, prepare, and act on current decisions (62). Such operations are essential parts of performing any type of cognitive control task in that a recent response outcome, whether it be a correct or error response, could influence how an individual performs on subsequent trials. Since only the SZ group showed suppression in this region, SZ-specific deficits could be related to these more sustained forms of processing which could compound with other error processing or performance monitoring deficits.

As with any study involving participants with psychiatric disorders, the effects of symptom severity and psychotropic medication could influence SZ-specific results. There were no significant associations, however, between activation in SZ-specific clusters and symptom subscale scores or CPZ equivalents (see Table S2 in Supplementary Material). Previous research also has failed to find such associations (30). Another possibility is that poor antisaccade performance in both LCC and SZ groups could be due to other underlying factors, like IQ, rather than poor cognitive control *per se*. IQ is highly associated with, and accurately predicted by working memory capacity (36, 63), the sole factor we used to establish our LCC and HCC groups. As in previous studies (14), the nature of this IQ/working memory capacity relationship extended to antisaccade performance in our sample; there was a significant positive correlation between the percentage of correct responses and scores on the Information scale from the Verbal Comprehension Subtests of the WAIS (a coarse approximation of IQ) [*r*(70) = 0.39, *p* = 0.0008]. While IQ and cognitive control may not be differentiable in this study, the antisaccade measures of performance and brain activation demonstrate that not all deficits are specific to SZ. Future studies with these groups should include larger sample sizes, as well as the inclusion of a group of people with SZ with preserved cognitive control. The latter would provide further insight into brain mechanisms underlying poor cognitive control in healthy people and people with SZ.

This study used two healthy comparison groups, people with HCC and people with LCC, to better understand which antisaccade deficits were specific to SZ and which were due to poor cognitive control abilities. When compared to the HCC group, both the LCC and SZ groups had poorer antisaccade performance and showed dysfunction of a distributed salience network composed of fronto-insular and cingulate regions during processing of correct and error responses. Strong overlap in behavior and brain between the LCC and SZ group should inform the selection of control groups in future neuroimaging studies. SZ-specific disruptions were evidenced by suppression of the STG which could indicate problems in sustained maintenance of outcome information to inform future behavior. Understanding the neural similarities and differences between groups that display similar behavioral performance may inform models of cognitive control, especially in disorders like SZ, where deficits in cognitive control are common, but treatment is limited.

## Ethics Statement

Study procedures were reviewed and approved by the University of Georgia Institutional Review Board. All subjects signed consent forms before participation in any aspects of the study.

## Author Contributions

AR, DS, BC, and JM conceived experiments. AR and DS carried out experiments. AR analyzed and interpreted data. AR wrote the manuscript. BC and JM reviewed and edited the manuscript.

## Conflict of Interest Statement

The authors declare that the research was conducted in the absence of any commercial or financial relationships that could be construed as a potential conflict of interest.

## References

[1] Hallett P Primary and secondary saccades to goals defined by instructions. Vision Res (1978) 18(10):1279–96. 10.1016/0042-6989(78)90218-3 726270

[2] Roberts RJ Hager LD Heron C Prefrontal cognitive processes: Working memory and inhibition in the antisaccade task. J Exp Psychol Gen (1994) 123(4):374 10.1037/0096-3445.123.4.374

[3] Unsworth N Schrock JC Engle RW. Working memory capacity and the antisaccade task: individual differences in voluntary saccade control. J Exp Psychol Learn Mem Cogn (2004) 30(6):1302. 10.1037/0278-7393.30.6.1302 15521806

[4] Berman RA Colby C Genovese C Voyvodic J Luna B Thulborn K Cortical networks subserving pursuit and saccadic eye movements in humans: an FMRI study. Hum Brain Mapp (1999) 8(4):209–25. 10.1002/(SICI)1097-0193(1999)8:4<209::AID-HBM5>3.0.CO;2-0 10619415 PMC6873313

[5] McDowell JE Brown GG Paulus M Martinez A Stewart SE Dubowitz DJ Neural correlates of refixation saccades and antisaccades in normal and schizophrenia subjects. Biol Psychiatry (2002) 51(3):216–23. 10.1016/S0006-3223(01)01204-5 11839364

[6] Matsuda T Matsuura M Ohkubo T Ohkubo H Matsushima E Inoue K Functional MRI mapping of brain activation during visually guided saccades and antisaccades: cortical and subcortical networks. Psychiatry Res (2004) 131(2):147–55. 10.1016/j.pscychresns.2003.12.007 15313521

[7] Ford KA Goltz HC Brown MR Everling S. Neural processes associated with antisaccade task performance investigated with event-related FMRI. J Neurophysiol (2005) 94(1):429–40. 10.1152/jn.00471.2004 15728770

[8] Camchong J Dyckman KA Austin BP Clementz BA McDowell JE. Common neural circuitry supporting volitional saccades and its disruption in schizophrenia patients and relatives. Biol Psychiatry (2008) 64(12):1042–50. 10.1016/j.biopsych.2008.06.015 18692173 PMC3339629

[9] McDowell JE Dyckman KA Austin BP Clementz BA. Neurophysiology and neuroanatomy of reflexive and volitional saccades: evidence from studies of humans. Brain Cogn (2008) 68(3):255–70. 10.1016/j.bandc.2008.08.016 18835656 PMC2614688

[10] Guitton D Buchtel H Douglas R. Frontal lobe lesions in man cause difficulties in suppressing reflexive glances and in generating goal-directed saccades. Exp Brain Res (1985) 58(3):455–72. 10.1007/BF00235863 4007089

[11] Fukushima J Fukushima K Miyasaka K Yamashita I. Voluntary control of saccadic eye movement in patients with frontal cortical lesions and parkinsonian patients in comparison with that in schizophrenics. Biol Psychiatry (1994) 36(1):21–30. 10.1016/0006-3223(94)90058-2 8080899

[12] Pierrot-Deseilligny C Müri R Rivaud S Gaymard B Eye movement disorders after prefrontal cortex lesions in humans. Society for Neuroscience Abstract. (1995). 1270 p.

[13] Pierrot-Deseilligny C Ploner C Müri R Gaymard B Rivaud-Pechoux S. Effects of cortical lesions on saccadic eye movements in humans. Ann N Y Acad Sci (2002) 956(1):216–29. 10.1111/j.1749-6632.2002.tb02821.x 11960806

[14] Evdokimidis I Smyrnis N Constantinidis T Stefanis N Avramopoulos D Paximadis C The antisaccade task in a sample of 2,006 young men. Exp Brain Res (2002) 147(1):45–52. 10.1007/s00221-002-1208-4 12373368

[15] McDowell JE Clementz BA. Behavioral and brain imaging studies of saccadic performance in schizophrenia. Biol Psychol (2001) 57(1–3):5–22. 10.1016/S0301-0511(01)00087-4 11454432

[16] Reuter B Kathmann N. Using saccade tasks as a tool to analyze executive dysfunctions in schizophrenia. Acta Psychol (Amst) (2004) 115(2–3):255–69. 10.1016/j.actpsy.2003.12.009 14962403

[17] Hutton SB Ettinger U The antisaccade task as a research tool in psychopathology: a critical review. Psychophysiology (2006) 43(3):302–13. 10.1111/j.1469-8986.2006.00403.x 16805870

[18] Clementz BA McDowell JE Zisook S. Saccadic system functioning among schizophrenia patients and their first-degree biological relatives. J Abnorm Psychol (1994) 103(2):277. 10.1037/0021-843X.103.2.400 8040497

[19] Sereno AB Holzman PS Antisaccades and smooth pursuit eye movements in schizophrenia. Biol Psychiatry (1995) 37(6):394–401. 10.1016/0006-3223(94)00127-O 7772648

[20] McDowell JE Myles-Worsley M Coon H Byerley W Clementz BA. Measuring liability for schizophrenia using optimized antisaccade stimulus parameters. Psychophysiology (1999) 36(1):138–41. 10.1017/S0048577299980836 10098389

[21] Raemaekers M Jansma JM Cahn W Van der Geest JN van der Linden JA Kahn RS Neuronal substrate of the saccadic inhibition deficit in schizophrenia investigated with 3-dimensional event-related functional magnetic resonance imaging. Arch Gen Psychiatry (2002) 59(4):313–20. 10.1001/archpsyc.59.4.313 11926931

[22] Ettinger U Picchioni M Hall MH Schulze K Toulopoulou T Landau S Antisaccade performance in monozygotic twins discordant for schizophrenia: the Maudsley twin study. Am J Psychiatry (2006) 163(3):543–5. 10.1176/appi.ajp.163.3.543 16513882

[23] Radant AD Dobie DJ Calkins ME Olincy A Braff DL Cadenhead KS Successful multi-site measurement of antisaccade performance deficits in schizophrenia. Schizophr Res (2007) 89(1):320–9. 10.1016/j.schres.2006.08.010 17023145

[24] Camchong J Dyckman KA Chapman CE Yanasak NE McDowell JE. Basal ganglia-thalamocortical circuitry disruptions in schizophrenia during delayed response tasks. Biol Psychiatry (2006) 60(3):235–41. 10.1016/j.biopsych.2005.11.014 16458267

[25] Keedy SK Ebens CL Keshavan MS Sweeney JA. Functional magnetic resonance imaging studies of eye movements in first episode schizophrenia: smooth pursuit, visually guided saccades and the oculomotor delayed response task. Psychiatry Res (2006) 146(3):199–211. 10.1016/j.pscychresns.2006.01.003 16571373

[26] Tu PC Yang TH Kuo WJ Hsieh JC Su TP. Neural correlates of antisaccade deficits in schizophrenia, an fMRI study. J Psychiatr Res (2006) 40(7):606–12. 10.1016/j.jpsychires.2006.05.012 16842821

[27] Ettinger U Ffytche DH Kumari V Kathmann N Reuter B Zelaya F Decomposing the neural correlates of antisaccade eye movements using event-related fMRI. Cereb Cortex (2008) 18(5):1148–59. 10.1093/cercor/bhm147 17728263

[28] Polli FE Barton JJS Thakkar KN Greve DN Goff DC Rauch SL Reduced error-related activation in two anterior cingulate circuits is related to impaired performance in schizophrenia. Brain (2008) 131(4):971–86. 10.1093/brain/awm307 18158315

[29] Ettinger U Kumari V Crawford TJ Corr PJ Das M Zachariah E Smooth pursuit and antisaccade eye movements in siblings discordant for schizophrenia. J Psychiatr Res (2004) 38(2):177–84. 10.1016/S0022-3956(03)00105-5 14757332

[30] Reilly JL Frankovich K Hill S Gershon ES Keefe RS Keshavan MS Elevated antisaccade error rate as an intermediate phenotype for psychosis across diagnostic categories. Schizophr Bull (2014) 40(5):1011–21. 10.1093/schbul/sbt132 24080895 PMC4133662

[31] Hutton SB Huddy V Barnes TR Robbins TW Crawford TJ Kennard C The relationship between antisaccades, smooth pursuit, and executive dysfunction in first-episode schizophrenia. Biol Psychiatry (2004) 56(8):553–9. 10.1016/j.biopsych.2004.07.002 15476684

[32] Dyckman KA Lee AKC Agam Y Vangel M Goff DC Barton JJS Abnormally persistent fMRI activation during antisaccades in schizophrenia: a neural correlate of perseveration? Schizophr Res (2011) 132(1):62–8. 10.1016/j.schres.2011.07.026 21831602 PMC3172368

[33] Kang SS Dionisio DP Sponheim SR. Abnormal mechanisms of antisaccade generation in schizophrenia patients and unaffected biological relatives of schizophrenia patients. Psychophysiology (2011) 48(3):350–61. 10.1111/j.1469-8986.2010.01074.x 20636287 PMC2994980

[34] Kane MJ Bleckley MK Conway AR Engle RW. A controlled-attention view of working-memory capacity. J Exp Psychol Gen (2001) 130(2):169. 10.1037/0096-3445.130.2.169 11409097

[35] Schaeffer DJ Amlung MT Li Q Krafft CE Austin BP Dyckman KA Neural correlates of behavioral variation in healthy adults’ antisaccade performance. Psychophysiology (2013) 50(4):325–33. 10.1111/psyp.12030 23418930 PMC13671259

[36] Conway AR Cowan N Bunting MF Therriault DJ Minkoff SR A latent variable analysis of working memory capacity, short-term memory capacity, processing speed, and general fluid intelligence. Intelligence (2002) 30(2):163–83. 10.1016/S0160-2896(01)00096-4

[37] Engle RW Tuholski SW Laughlin JE Conway AR. Working memory, short-term memory, and general fluid intelligence: a latent-variable approach. J Exp Psychol Gen (1999) 128(3):309. 10.1037/0096-3445.128.3.309 10513398

[38] Unsworth N Engle RW. The nature of individual differences in working memory capacity: active maintenance in primary memory and controlled search from secondary memory. Psychol Rev (2007) 114(1):104. 10.1037/0033-295X.114.1.104 17227183

[39] Unsworth N Spillers GJ Brewer GA. The role of working memory capacity in autobiographical retrieval: individual differences in strategic search. Memory (2012) 20(2):167–76. 10.1080/09658211.2011.651087 22273543

[40] Cox RW. AFNI: software for analysis and visualization of functional magnetic resonance neuroimages. Comput Biomed Res (1996) 29(3):162–73. 10.1006/cbmr.1996.0014 8812068

[41] DyckmanKACamchongJClementzBAMcDowellJE. An effect of context on saccade-related behavior and brain activity. Neuroimage (2007) 36(3):774–84.10.1016/j.neuroimage.2007.03.02317478104

[42] Chen G Saad ZS Nath AR Beauchamp MS Cox RW. FMRI group analysis combining effect estimates and their variances. Neuroimage (2012) 60(1):747–65. 10.1016/j.neuroimage.2011.12.060 22245637 PMC3404516

[43] Crawford TJ Puri B Nijran K Jones B Kennard C Lewis S. Abnormal saccadic distractibility in patients with schizophrenia: a 99m Tc-HMPAO SPET study. Psychol Med (1996) 26(02):265–77. 10.1017/S0033291700034668 8685283

[44] Menon V Uddin LQ. Saliency, switching, attention and control: a network model of insula function. Brain Struct Funct (2010) 214(5):655–67. 10.1007/s00429-010-0262-0 20512370 PMC2899886

[45] Dosenbach NUF Visscher KM Palmer ED Miezin FM Wenger KK Kang HC A core system for the implementation of task sets. Neuron (2006) 50(5):799–812. 10.1016/j.neuron.2006.04.031 16731517 PMC3621133

[46] Corbetta M Shulman GL. Control of goal-directed and stimulus-driven attention in the brain. Nat Rev Neurosci (2002) 3(3):201–15. 10.1038/nrn755 11994752

[47] Eckert MA Menon V Walczak A Ahlstrom J Denslow S Horwitz A At the heart of the ventral attention system: the right anterior insula. Hum Brain Mapp (2009) 30(8):2530–41. 10.1002/hbm.20688 19072895 PMC2712290

[48] Shulman GL Astafiev SV Franke D Pope DLW Snyder AZ McAvoy MP Interaction of stimulus-driven reorienting and expectation in ventral and dorsal fronto-parietal and basal ganglia-cortical networks. J Neurosci (2009) 29(14):4392–407. 10.1523/JNEUROSCI.5609-08.2009 19357267 PMC2743562

[49] Aron AR Robbins TW Poldrack RA. Inhibition and the right inferior frontal cortex. Trends Cogn Sci (2004) 8(4):170–7. 10.1016/j.tics.2004.02.010 15050513

[50] Duann J-R Ide JS Luo X Li C-SR. Functional connectivity delineates distinct roles of the inferior frontal cortex and presupplementary motor area in stop signal inhibition. J Neurosci (2009) 29(32):10171–9. 10.1523/jneurosci.1300-09.2009 19675251 PMC2769086

[51] Verbruggen F Aron AR Stevens MA Chambers CD. Theta burst stimulation dissociates attention and action updating in human inferior frontal cortex. Proc Natl Acad Sci U S A (2010) 107(31):13966–71. 10.1073/pnas.1001957107 20631303 PMC2922216

[52] Dickinson D Ramsey ME Gold JM. Overlooking the obvious: a meta-analytic comparison of digit symbol coding tasks and other cognitive measures in schizophrenia. Arch Gen Psychiatry (2007) 64(5):532–42. 10.1001/archpsyc.64.5.532 17485605

[53] Vossel S Geng JJ Fink GR Dorsal and ventral attention systems. Neuroscientist (2013) 20(2):150–9. 10.1177/1073858413494269 23835449 PMC4107817

[54] Ham T Leff A de Boissezon X Joffe A Sharp DJ. Cognitive control and the salience network: an investigation of error processing and effective connectivity. J Neurosci (2013) 33(16):7091–8. 10.1523/JNEUROSCI.4692-12.2013 23595766 PMC6618896

[55] Carter CS MacDonald AW III Ross LL Stenger VA. Anterior cingulate cortex activity and impaired self-monitoring of performance in patients with schizophrenia: an event-related fMRI study. Am J Psychiatry (2001) 158(9):1423–8. 10.1176/appi.ajp.158.9.1423 11532726

[56] Laurens KR Ngan ET Bates AT Kiehl KA Liddle PF Rostral anterior cingulate cortex dysfunction during error processing in schizophrenia. Brain (2003) 126(3):610–22. 10.1093/brain/awg056 12566282

[57] Kerns JG Cohen JD MacDonald AW III Johnson MK Stenger VA Aizenstein H Decreased conflict-and error-related activity in the anterior cingulate cortex in subjects with schizophrenia. Am J Psychiatry (2005) 162(10):1833–9. 10.1176/appi.ajp.162.10.1833 16199829

[58] Mesulam MM Mufson EJ. Insula of the old world monkey. III: efferent cortical output and comments on function. J Comp Neurol (1982) 212(1):38–52. 10.1002/cne.902120104 7174907

[59] Mufson EJ Mesulam MM. Insula of the old world monkey. II: afferent cortical input and comments on the claustrum. J Comp Neurol (1982) 212(1):23–37. 10.1002/cne.902120103 7174906

[60] Augustine JR. The insular lobe in primates including humans. Neurol Res (1985) 7(1):2–10. 10.1080/01616412.1985.11739692 2860583

[61] Chang LJ Yarkoni T Khaw MW Sanfey AG. Decoding the role of the insula in human cognition: functional parcellation and large-scale reverse inference. Cereb Cortex (2013) 23(3):739–49. 10.1093/cercor/bhs065 22437053 PMC3563343

[62] Paulus MP Feinstein JS Leland D Simmons AN. Superior temporal gyrus and insula provide response and outcome-dependent information during assessment and action selection in a decision-making situation. Neuroimage (2005) 25(2):607–15. 10.1016/j.neuroimage.2004.12.055 15784440

[63] Conway ARA Kane MJ Engle RW. Working memory capacity and its relation to general intelligence. Trends Cogn Sci (2003) 7(12):547–52. 10.1016/j.tics.2003.10.005 14643371

