# Supplementary material for: Evaluating the Specificity of Cognitive Control Deficits in Schizophrenia Using Antisaccades, Functional Magnetic Resonance Imaging, and Healthy Individuals With Poor Cognitive Control
Source: Front Psychiatry. 2018 Apr 11;9:107. doi: 10.3389/fpsyt.2018.00107 (PMC5904188; doi:10.3389/fpsyt.2018.00107)
Supplement: Supplementary file 1 [file Table_1.DOCX]

| **Supplemental Table 1.** Results of One-Way ANOVAs Testing Differences in Stimulus-Correlated Movement | | |
| --- | --- | --- |
| Motion Parameter | Run 1 | Run 2 |
| Roll | F(2,68) = .65, *p =* .52 | F(2,68) = .50, *p =* .60 |
| Pitch | F(2,68) = .09, *p =* .91 | F(2,68) = .08, *p =* .92 |
| Yaw | F(2,68) = 1.48, *p =* .23 | F(2,68) = 2.04, *p =* .13 |
| Shift X | F(2,68) = .38 , *p =* .68 | F(2,68) = .26, *p =* .77 |
| Shift Y | F(2,68) = .72 , *p =* .49 | F(2,68) = .38, *p =* .68 |
| Shift Z | F(2,68) = .37 , *p =* .69 | F(2,68) = 1.64, *p =* .20 |
| ^a^Amount of stimulus-correlated movement in each subject was quantified by correlating motion parameters (obtained from the alignment step during subject-level preprocessing) with the antisaccade reference function for each run. A Fisher’s Z transform was applied to the correlations, and one-way ANOVAs were used to test the difference in stimulus-correlated movement across the three groups (HCC, LCC, SZ) for each motion parameter. There were no significant differences in stimulus-correlated movement among the three groups for any parameter. | | |
