# Supplementary material for: Evaluating the Specificity of Cognitive Control Deficits in Schizophrenia Using Antisaccades, Functional Magnetic Resonance Imaging, and Healthy Individuals With Poor Cognitive Control
Source: Front Psychiatry. 2018 Apr 11;9:107. doi: 10.3389/fpsyt.2018.00107 (PMC5904188; doi:10.3389/fpsyt.2018.00107)
Supplement: Supplementary file 2 [file Table_2.DOCX]

| **Supplement Table 2.** Correlations between Symptom Ratings, CPZ Daily Dose and STG/Insula Activation during Error Trials | | | | | | | |
| --- | --- | --- | --- | --- | --- | --- | --- |
|  | PANSS pos |  | PANSS neg |  | PANSS gen |  | CPZ Daily Dose Equivalent |
| Right STG/Insula | .06 |  | .14 |  | -.0006 |  | -.27 |
| Left STG/Insula | -.16 |  | .31 |  | -.05 |  | -.11 |
| ^a^Table shows Pearson correlation coefficients between activation values in the schizophrenia group and PANSS sub-test scores (n = 20) and CPZ daily dose equivalent (n=10) for clusters that showed schizophrenia-specific deficits in activation during antisaccade error trials. No correlation coefficients were significant at *p* < .05. pos = positive; neg = negative; gen = general; PANSS = Positive and Negative Symptom Scale; CPZ = chlorpromazine; STG = superior temporal gyrus. | | | | | | | |
